# Post-surgical Pyoderma Gangrenosum Reveals Celiac Disease in a Pediatric Patient

**DOI:** 10.7759/cureus.87137

**Published:** 2025-07-01

**Authors:** Zahidul Islam, Daniel Alicea, Nicole Schiraldi, Adam Chahine, David Ciocon

**Affiliations:** 1 Dermatology, New York Medical College, New York, USA; 2 Dermatology, Albert Einstein College of Medicine, New York, USA; 3 Dermatology, Downstate Health Sciences University, New York, USA

**Keywords:** autoinflammatory, celiac disease, inflammatory skin condition, pathergy, pediatric dermatology, pediatrics, post-surgical, pyoderma gangrenosum

## Abstract

Pyoderma gangrenosum (PG) is a rare, autoinflammatory neutrophilic dermatosis that may be triggered by cutaneous trauma and is frequently associated with systemic diseases, most commonly inflammatory bowel disease. Celiac disease (CD), an immune-mediated enteropathy precipitated by gluten, is not typically linked with PG, particularly in the pediatric population. We present a highly unusual case of a 13-year-old female who developed post-surgical PG following excision of an epidermoid cyst, ultimately leading to a new diagnosis of CD. Histopathology and clinical features were consistent with PG, with no evidence of infection. The patient was successfully managed with topical corticosteroids and wound care. Subsequent gastrointestinal evaluation revealed positive celiac serologies and confirmatory duodenal biopsy findings. This case highlights the importance of recognizing PG as a potential cutaneous manifestation of undiagnosed CD in pediatric patients and the need for a multidisciplinary approach to prompt diagnosis and treatment.

## Introduction

Pyoderma gangrenosum (PG) is an inflammatory skin disorder characterized by painful, rapidly progressing ulcers and is considered a diagnosis of exclusion [1]. Although its pathogenesis is complex and not fully elucidated, PG is hypothesized to involve dysregulated inflammatory mediators and an excessive neutrophil and T-cell response [2]. It is frequently associated with systemic conditions such as inflammatory bowel disease (IBD), rheumatologic diseases, and hematologic malignancies, with IBD being the most common. Minor trauma or surgical procedures can induce PG through pathergy [1].

Celiac disease (CD) is an autoimmune disorder of the gastrointestinal tract triggered by gluten consumption. The resulting autoinflammatory response leads to small intestinal damage and systemic manifestations. It affects approximately 1% of children [3]. While PG is associated with many autoimmune conditions, its link to CD is rare. We present a unique case of postsurgical PG in a pediatric patient with previously undiagnosed CD. To our knowledge, only two prior case reports have documented this association [4,5]. Given that PG accounts for fewer than 5% of cases in pediatric populations, this case highlights an unusual and novel connection between PG and CD [1].

## Case presentation

**Figure 1:**
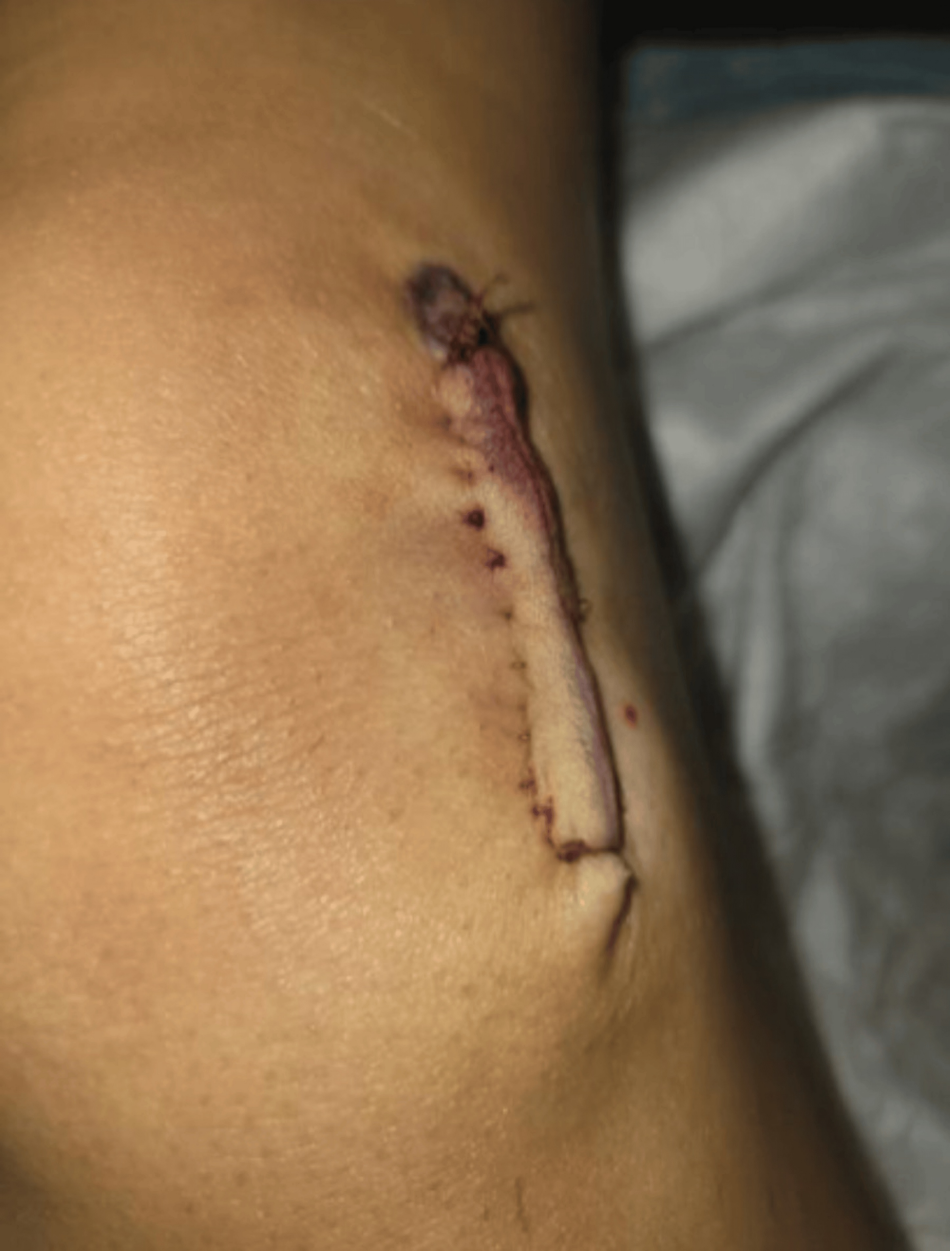
The surgical site after removal of EIC on postoperative day one EIC: Epidermoid cyst

A 13-year-old female with no significant past medical or family history, allergies, or previous surgeries presented to our dermatologic surgery clinic for the removal of an epidermoid cyst (EIC). The lesion, located on the left lateral knee, had gradually enlarged over five years. It measured 2.5 × 2.5 cm and was surgically excised without intraoperative complications. Figure 1 shows the surgical site one day after the procedure, with no abnormal findings.

**Figure 2:**
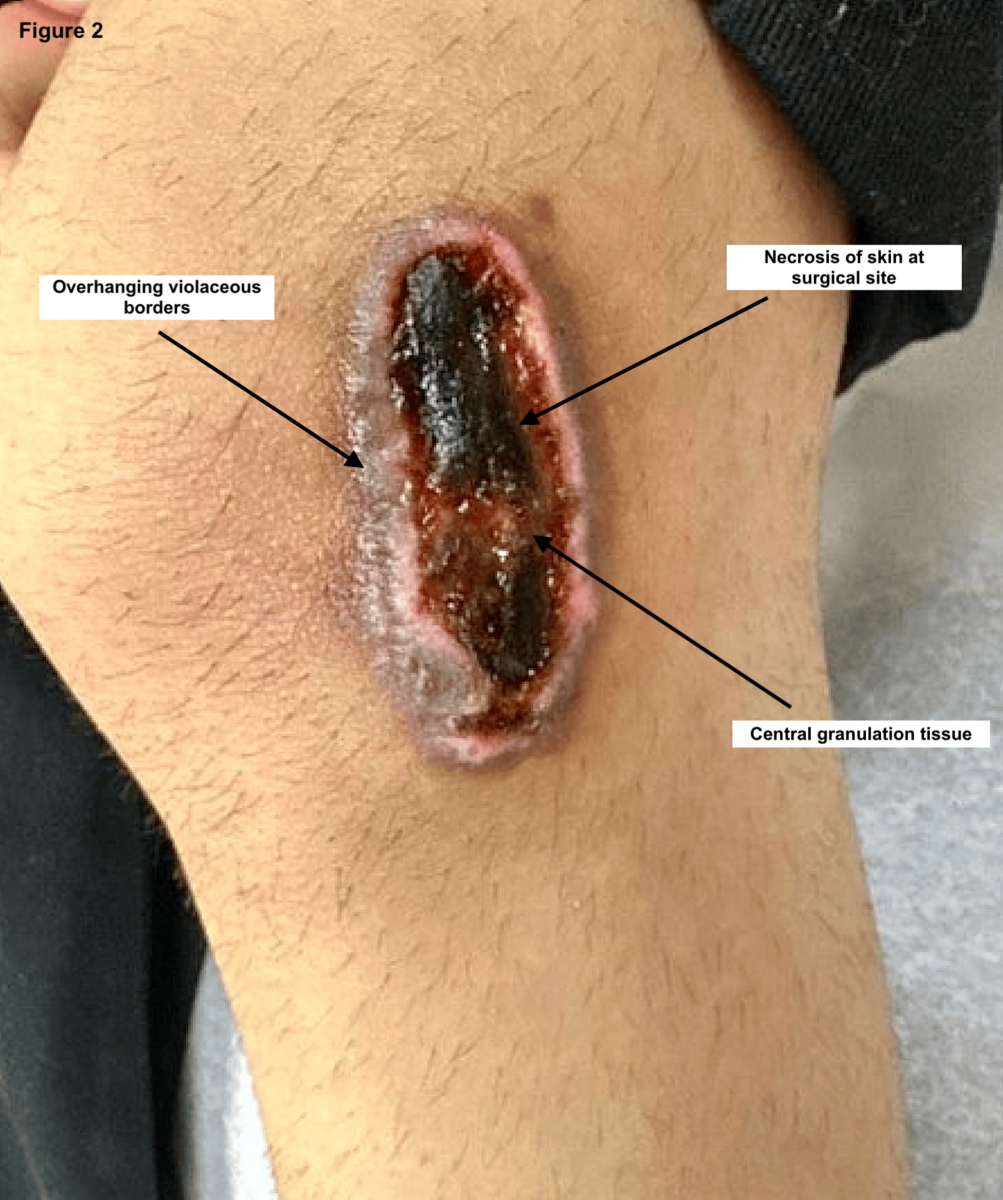
Surgical site on postoperative day 35 Seen in the picture is the 9 cm × 4 cm ulcerated wound with overhanging violaceous borders, central granulation tissue, and necrosis.

**Figure 3:**
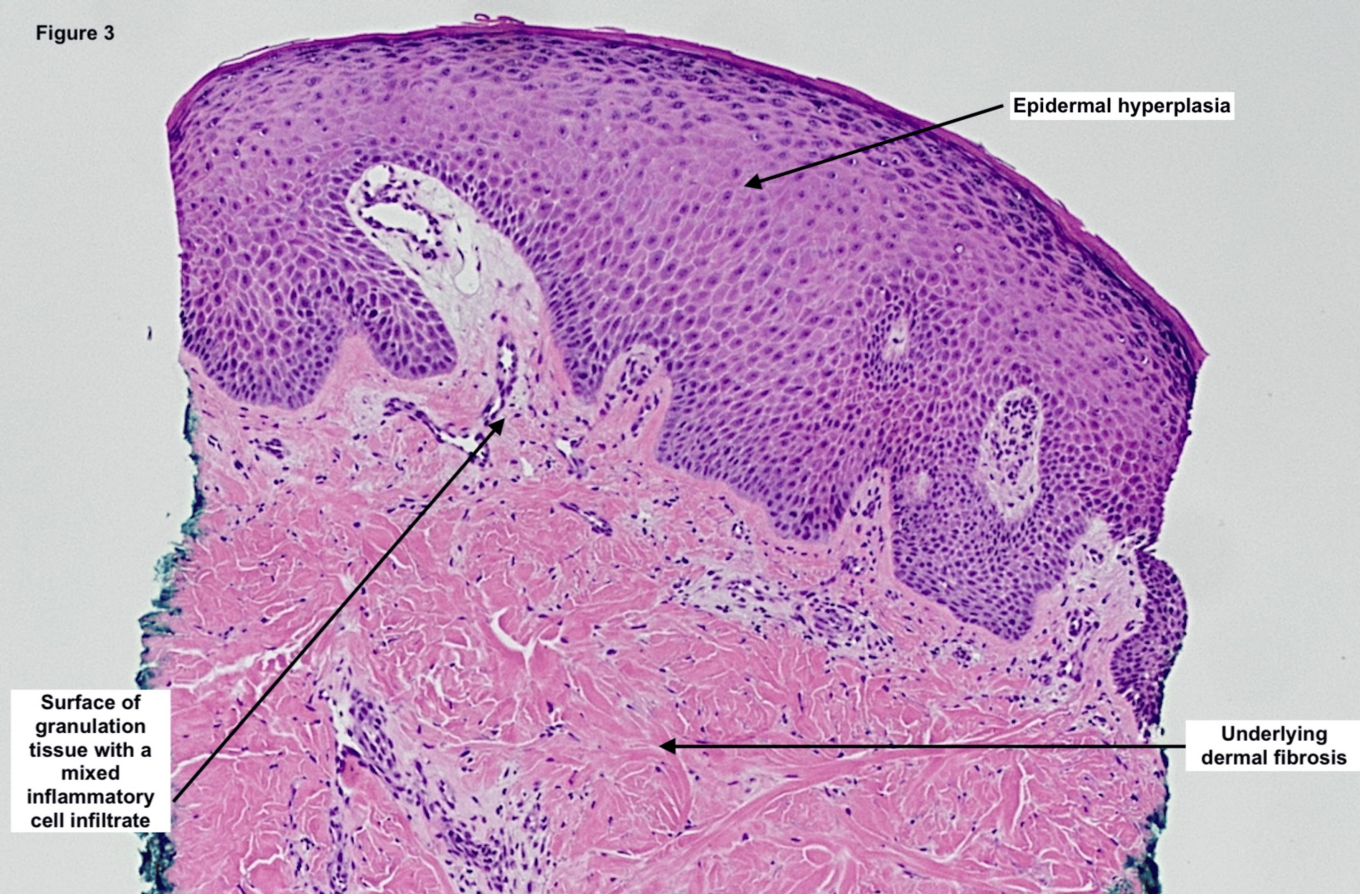
Hematoxylin and eosin stain of punch biopsy taken at the PG lesion border The histopathologic examination shows epidermal hyperplasia, underlying dermal fibrosis, and a surface of granulation tissue with a mixed inflammatory cell infiltrate.

Four days postoperatively, the patient’s mother noted increasing pain, violaceous discoloration, and wound breakdown. By day 21, the surgical site had progressed into a necrotic, enlarging ulcer. By day 25, the lesion exhibited overhanging violaceous borders and central purulence. At the follow-up visit on postop day 35, clinical features were consistent with PG (Figure 2). The ulcer measured approximately 9 × 4 cm. A punch biopsy from the lesion edge revealed epidermal hyperplasia, dermal fibrosis, and granulation tissue with mixed inflammatory infiltrate; stains for fungal, bacterial, and acid-fast bacilli were negative (Figure 3). Wound cultures were not obtained due to the absence of drainage, fever, odor, warmth, or systemic signs of infection. Treatment was initiated with triamcinolone acetonide ointment, collagenase ointment, and topical gentamicin. Gentamicin was used empirically in the early course out of concern for potential secondary infection. The patient experienced rapid clinical improvement.

During follow-up, additional history revealed that the patient experienced recurrent abdominal pain after consuming gluten-containing foods, previously misattributed to menstrual cramps. She exhibited no signs of dermatitis herpetiformis. Pediatric gastroenterology evaluation showed normal labs (CBC, CMP, thyroid, autoimmune markers, hemoccult, calprotectin), but elevated celiac serologies: tissue transglutaminase IgA and deamidated gliadin peptide IgG. Duodenal biopsy confirmed CD with mild-to-moderate villous blunting and increased intraepithelial lymphocytes. At follow-up two months from symptom onset, the ulcerative PG lesion had completely resolved with only topical therapy. She was started on a gluten-free diet with nutritional guidance.

## Discussion

Pyoderma gangrenosum is a rare, non-infectious, autoinflammatory dermatosis characterized by the rapid onset of painful, ulcerative, and necrolytic skin lesions. Although traditionally classified as a neutrophilic dermatosis, recent evidence suggests that dysregulated T-cell responses targeting pilosebaceous units may also contribute to its pathogenesis [2]. Trauma-induced immune dysregulation, including pathergy, an exaggerated inflammatory response to minor trauma, is observed in approximately one-third of PG cases [2]. In our patient, PG was likely triggered by the surgical excision of the EIC.

Pyoderma gangrenosum is frequently associated with systemic immune-mediated diseases such as inflammatory bowel disease (e.g., Crohn’s disease and ulcerative colitis), rheumatoid arthritis, and hematologic malignancies. Its association with CD, however, is exceedingly rare. To date, only two adult cases have been reported [4,5], and, to our knowledge, this is the first reported case of pediatric PG associated with CD. Notably, our patient developed PG prior to her CD diagnosis, despite a longstanding history of gluten-related abdominal symptoms.

The immunopathogenesis linking PG and CD may be better understood within the broader spectrum of gluten-related immune-mediated skin disorders. Dermatitis herpetiformis (DH), the most well-characterized neutrophilic dermatosis associated with CD, shares immunopathogenic features with PG, including IL-8 and IL-15-driven neutrophilic inflammation [6,7]. Both PG and DH involve immune dysregulation related to HLA-DQ2 and HLA-DQ8 haplotypes. In DH, IgA deposits targeting tissue transglutaminase (TG2) and epidermal transglutaminase (TG3) contribute to neutrophil recruitment. Recent studies also suggest that gluten sensitivity may contribute to other inflammatory dermatoses such as psoriasis, linear IgA bullous dermatosis, and palmoplantar pustulosis, some of which improve with gluten-free diets [2,6-8]. These overlapping immune mechanisms lend plausibility to the rare but biologically feasible association between PG and CD.

Although PG is ultimately a clinical diagnosis of exclusion, histopathologic findings can help support the diagnosis. In our case, the biopsy showed epidermal hyperplasia, dermal fibrosis, and a mixed inflammatory infiltrate, while special stains for bacteria, fungi, and acid-fast bacilli were negative. Though the absence of wound cultures is a limitation, the lack of systemic signs of infection and the rapid clinical improvement with corticosteroids strongly support a sterile inflammatory etiology. The empiric use of gentamicin was discontinued once infection became unlikely. Several ulcerative dermatoses were considered in the differential diagnosis, including infectious ulcers, vasculitis, and Langerhans cell histiocytosis (LCH). Infectious ulcers were ruled out based on clinical stability, absence of fever or leukocytosis, and negative histochemical stains. Vasculitic ulcers often present with palpable purpura or livedo reticularis and demonstrate vessel wall inflammation and fibrinoid necrosis histologically, features that are not present here. Langerhans cell histiocytosis, though capable of producing ulcerative skin lesions in children, typically presents at a younger age and is characterized by histiocytes with grooved nuclei and positive CD1a or Langerin staining, which were absent in this case [9,10].

Treatment of PG typically begins with topical or systemic corticosteroids and may escalate to immunosuppressants (e.g., cyclosporine) or biologic therapies (e.g., TNF-α inhibitors) for refractory or extensive disease [1]. Treatment should be individualized based on disease severity, lesion extent, and presence of comorbidities such as Crohn’s or celiac disease, which may influence both therapeutic response and recurrence risk. In this case, the lesion remained localized, and the patient was otherwise well, allowing for successful treatment with high-potency topical corticosteroids, collagenase, and wound care alone. Systemic immunosuppressive therapy was not required due to the rapid and sustained response to topical management.

## Conclusions

This case demonstrates PG in a pediatric patient and its rare association with undiagnosed CD. Clinicians should maintain a high index of suspicion for underlying systemic disease in patients presenting with rapidly evolving ulcers post-trauma or surgery. Although wound cultures were not obtained, a limitation of this case, the combination of negative histologic stains and rapid topical steroid response supported a sterile process. This case illustrates how PG may be the initial clue to an underlying systemic autoimmune disorder. Pediatric patients with PG should be evaluated broadly, including gastrointestinal symptoms, to ensure timely diagnosis and treatment. Further studies are warranted to explore the pathophysiologic links and optimize treatment strategies in such rare associations.
